# An Investigation into the Potential of Targeting *Escherichia coli rne* mRNA with Locked Nucleic Acid (LNA) Gapmers as an Antibacterial Strategy

**DOI:** 10.3390/molecules26113414

**Published:** 2021-06-04

**Authors:** Layla R. Goddard, Charlotte E. Mardle, Hassan Gneid, Ciara G. Ball, Darren M. Gowers, Helen S. Atkins, Louise E. Butt, Jonathan K. Watts, Helen A. Vincent, Anastasia J. Callaghan

**Affiliations:** 1 School of Biological Sciences and Institute of Biological & Biomedical Sciences, University of Portsmouth, Portsmouth PO1 2DY, UK; layla.goddard@port.ac.uk (L.R.G.); charlotte.mardle@port.ac.uk (C.E.M.); ciara.ball@port.ac.uk (C.G.B.); darren.gowers@port.ac.uk (D.M.G.); louise.butt@port.ac.uk (L.E.B.); 2 Centre for Enzyme Innovation, University of Portsmouth, Portsmouth PO1 2DY, UK; 3 RNA Therapeutics Institute, University of Massachusetts Medical School, Worcester, MA 01609, USA; hgneid@tulane.edu (H.G.); Jonathan.Watts@umassmed.edu (J.K.W.); 4 Department of Chemistry, University of Southampton, Southampton SO17 1BJ, UK; 5 Defence Science and Technology Laboratory, Porton Down, Salisbury SP4 0JQ, UK; hsatkins@mail.dstl.gov.uk; 6 College of Life and Environmental Sciences, University of Exeter, Exeter EX4 4QD, UK

**Keywords:** antibacterial, antisense oligonucleotide, gapmer, locked nucleic acid (LNA), RNase E, RNase H, *rne* mRNA, translation blocking

## Abstract

The increase in antibacterial resistance is a serious challenge for both the health and defence sectors and there is a need for both novel antibacterial targets and antibacterial strategies. RNA degradation and ribonucleases, such as the essential endoribonuclease RNase E, encoded by the *rne* gene, are emerging as potential antibacterial targets while antisense oligonucleotides may provide alternative antibacterial strategies. As *rne* mRNA has not been previously targeted using an antisense approach, we decided to explore using antisense oligonucleotides to target the translation initiation region of the *Escherichia coli rne* mRNA. Antisense oligonucleotides were rationally designed and were synthesised as locked nucleic acid (LNA) gapmers to enable inhibition of *rne* mRNA translation through two mechanisms. Either LNA gapmer binding could sterically block translation and/or LNA gapmer binding could facilitate RNase H-mediated cleavage of the *rne* mRNA. This may prove to be an advantage over the majority of previous antibacterial antisense oligonucleotide approaches which used oligonucleotide chemistries that restrict the mode-of-action of the antisense oligonucleotide to steric blocking of translation. Using an electrophoretic mobility shift assay, we demonstrate that the LNA gapmers bind to the translation initiation region of *E. coli rne* mRNA. We then use a cell-free transcription translation reporter assay to show that this binding is capable of inhibiting translation. Finally, in an in vitro RNase H cleavage assay, the LNA gapmers facilitate RNase H-mediated mRNA cleavage. Although the challenges of antisense oligonucleotide delivery remain to be addressed, overall, this work lays the foundations for the development of a novel antibacterial strategy targeting *rne* mRNA with antisense oligonucleotides.

## 1. Introduction

The emergence of both natural and engineered antimicrobial resistant strains of bacteria poses a significant challenge to the health and defense sectors. Unfortunately, traditional drug development programmes, that successfully provided the antibiotics of the 20th century, are failing to keep pace with emerging resistance [1,2]. Consequently, there is a growing need for the development of novel antibacterial strategies that target alternative pathways and/or have unconventional mechanisms of action.

RNA degradation pathways and ribonucleases (RNases), the enzymes responsible for RNA turnover, have recently been identified as targets that could be exploited for antibiotic development [3,4]. Specifically, the endoribonuclease RNase E, and the *rne* gene that encodes it, are ideal candidates for antibacterial targeting for a number of reasons [3,4]. Firstly, RNase E/*rne* is essential [5,6,7,8] and so inhibitors of RNase E, or repressors of *rne* gene expression, would be expected to have antibacterial activity. Furthermore, RNase E/*rne* is implicated in bacterial virulence of the pathogens *Salmonella enterica* and *Yersinia pestis* [9,10]. Finally, RNase E/*rne* is highly conserved amongst Gram-negative bacteria but there is no known human orthologue [3,11] suggesting that specific inhibitors or repressors would target RNase E/*rne*-containing bacteria but not human hosts.

Some progress has been made in validating RNase E/*rne* as an antibacterial target through the identification of small molecule inhibitors of RNase E, using structure-based virtual high-throughput screening, and the characterisation of their inhibitory activity in vitro. Through this approach, a number of small molecules have been identified that inhibit RNase E from multiple bacterial pathogens in vitro [12,13]. However, the half maximal inhibitory concentration (IC_50_) for each of these inhibitors was in the low millimolar range, much higher than would be desired for an effective antibiotic [12,13]. Even enhanced inhibition, obtained using a combination of inhibitory small molecules, required millimolar concentrations of inhibitors [12]. Therefore, while these small molecules have potential as lead compounds for the development of antibiotics targeting RNase E, there is work still to be done.

An alternative antibacterial strategy to using small molecule antibiotics is the development of antisense oligonucleotide antibacterials reviewed in [14,15,16,17]. Antisense oligonucleotides are short, single-stranded nucleic acid sequences that are complementary to a target mRNA. They can down-regulate gene expression by binding to their target mRNA and inhibiting its translation through the creation of a steric block to ribosome binding and/or by facilitating RNase H recruitment and RNA cleavage [14,15,16,17] (Supplementary Figure S1). Typically, antisense oligonucleotides are synthesised from nucleotide analogues (Supplementary Figure S2) in order to enhance the affinity for RNA and decrease the susceptibility to cellular nucleases (reviewed in [14,15,16,17,18]). However, these chemical modifications can also negatively affect RNase H recruitment and limit the mode-of-action of the antisense oligonucleotide to steric blocking of ribosome binding [17,18]. A key advantage of the antisense approach is that it should be possible to rationally design an antisense oligonucleotide to target any mRNA. If the target mRNA encodes an essential protein, e.g., *rne* mRNA, then the antisense oligonucleotide may have antibacterial properties. A number of antisense oligonucleotides, that target a variety of mRNAs, have been reported to have antibacterial activity (see Supplementary Table S1 for examples).

In the current study we explored the potential of targeting *rne* mRNA with antisense oligonucleotides as a possible alternative antibacterial strategy. To our knowledge, *rne* mRNA has not been previously targeted using an antisense approach. We rationally designed two oligonucleotide sequences to have complementarity to the translation initiation region of *Escherichia coli rne* mRNA. Both sequences were synthesised as locked nucleic acid (LNA) gapmers, oligonucleotides consisting of a central region of DNA flanked by regions of chemically modified LNA nucleotides [18], with an LNA_3_-DNA_10_-LNA_3_ and an LNA_4_-DNA_8_-LNA_4_ configuration. The ability of each of the four LNA gapmers to bind to the translation initiation region of *E. coli rne* mRNA, inhibit translation, and recruit RNase H to mediate mRNA cleavage, was evaluated in vitro using an electrophoretic mobility shift assay (EMSA), a cell-free reporter assay and a gel-based RNase H cleavage assay, respectively. All four of the LNA gapmers bound to the translation initiation region of *E. coli rne* mRNA, inhibited translation and facilitated RNase H-mediated cleavage. However, there were preferences with regard to the antisense oligonucleotide sequence/binding site and gapmer configuration. These studies clearly demonstrate that it is possible to target *rne* mRNA with antisense oligonucleotides and they provide key knowledge that could be taken forwards to develop a novel antibacterial strategy.

## 2. Results

### 2.1. Targeting E. coil rne with LNA Gapmers

The first step in investigating an antisense approach to potentially down-regulate *rne* gene expression was to identify a region of the *E. coli rne* mRNA to target and rationally design antisense oligonucleotides against it. Most bacterial antisense oligonucleotides target the translation initiation region of an mRNA [17]. This is because this region of an mRNA is usually unstructured and accessible to ribosomes meaning that it will likely also be accessible to an antisense oligonucleotide [17]. In addition, antisense oligonucleotide binding to this region of the mRNA is most likely to sterically block translation by preventing ribosome binding which may be more effective than a steric block aimed at halting ribosome progression at a downstream binding site. Indeed, although *E. coli rne* mRNA contains a long (361-nucleotide), highly structured 5′ UTR, the translation initiation region has been reported to be unstructured [19]. Therefore we decided to target the translation initiation region of *E. coli rne* mRNA with antisense oligonucleotides.

**Figure 1:**
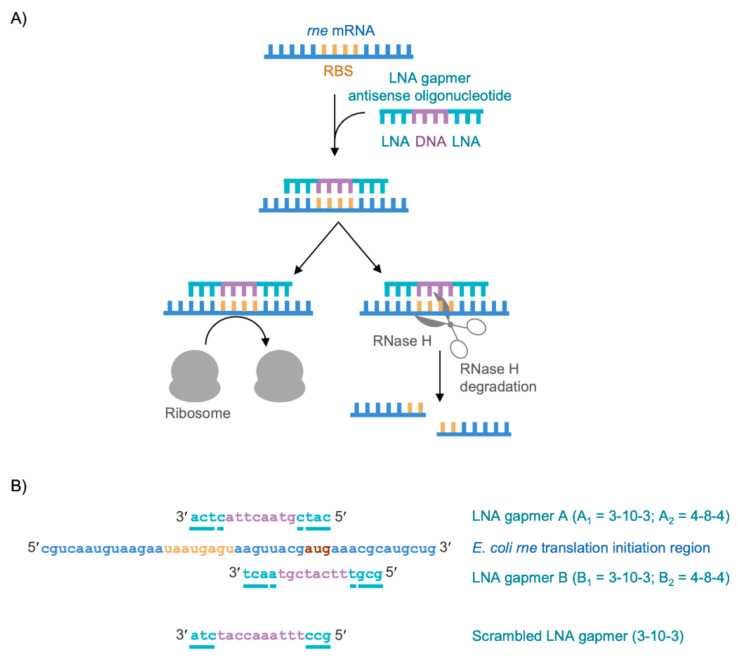
Targeting the translation initiation region of *rne* mRNA with LNA gapmers. (**A**) An antisense LNA gapmer comprising DNA (mauve) flanked by LNA (teal) binds to the translation initiation region (including the ribosome binding site (RBS), gold) of *rne* mRNA (blue). This prevents the ribosome (grey) from binding and sterically blocks translation. It also recruits RNase H (grey scissors) which cleaves the *rne* mRNA to prevent RNase E synthesis. (**B**) LNA gapmer A and LNA gapmer B were designed to be complementary to the translation initiation region of *E. coli rne* mRNA (blue; RBS, gold; start codon, brown). Scrambled LNA gapmer, which is not complementary to the translation initiation region of *E. coli rne* mRNA, was also designed. In each of the LNA gapmers, LNA bases (teal/underlined) flank DNA bases (mauve). Each LNA gapmer was synthesised as an LNA_3_-DNA_10_-LNA_3_ 3-10-3 gapmer (LNA gapmer A_1_/B_1_ and Scrambled LNA gapmer). LNA gapmers A and B were also synthesised as an LNA_4_-DNA_8_-LNA_4_ 4-8-4 gapmer (LNA gapmer A_2_/B_2_).

Next, we needed to select a suitable antisense oligonucleotide chemistry. Chemical analogues that are commonly used in bacterial antisense oligonucleotide chemistry include phosphorothioate, phosphorodiamidate morpholino (PMO), peptide nucleic acid (PNA) and LNA (Supplementary Figure S2) [17,18]. Phosphorothioate has a reduced affinity for RNA, compared to DNA, but phosphorothioate oligonucleotide:mRNA duplexes are recognised by RNase H [17,18]. In contrast, PMO, PNA and LNA all have a significantly greater affinity for RNA but neither PMO oligonucleotide:mRNA, PNA oligonucleotide:mRNA nor LNA oligonucleotide:mRNA duplexes are recognised by RNase H [17,18]. The mode-of-action of entirely PMO, PNA or LNA antisense oligonucleotides is therefore restricted to steric blocking of translation. To overcome this possible limitation, gapmer antisense oligonucleotides consisting of a central region of DNA flanked by chemically modified nucleotides can be used [17,18]. The central DNA region of the gapmer facilitates RNase H recruitment and mRNA cleavage while the flanking chemically modified nucleotides provide the enhanced oligonucleotide stability and RNA binding affinity. In order to allow us to compare both the steric blocking of ribosome binding and the RNase H-mediated mRNA cleavage mode-of-action of antisense oligonucleotides, we decided to use LNA gapmers to target *rne* mRNA. This strategy is shown schematically in Figure 1A.

Finally, we needed to rationally design our antisense oligonucleotide sequence and decide on the LNA gapmer configurations to synthesise. Kurreck et al., found that LNA gapmers with a central region of phosphorothioate DNA of at least seven nucleotides were sufficient to facilitate recruitment of RNase H [20]. In addition, flanking LNA regions of three nucleotides were sufficient to increase gapmer binding affinity for RNA and, together with a phosphorothioate backbone, confer protection from nucleases [20]. Considering these parameters, two 16-mer sequences, LNA gapmer A and LNA gapmer B, were designed to be complementary to the translation initiation region of *rne* mRNA (Figure 1B). A non-complementary scrambled 16-mer sequence, Scrambled LNA gapmer, was also designed to use as a control (Figure 1B). LNA gapmer A would be expected to occlude both the ribosome binding site (RBS) and the start codon of the *rne* mRNA and would be expected to prevent ribosome binding. LNA gapmer B would only be expected to occlude the *rne* mRNA start codon. Binding of LNA gapmer B may, or may not, prevent ribosome binding but it would be expected to block the progression of bound ribosomes. Therefore comparing the activity of LNA gapmer A and LNA gapmer B may indicate whether there is a preferred antisense oligonucleotide binding site within the *rne* mRNA translation initiation region. Each LNA gapmer sequence was synthesised as an LNA_3_-DNA_10_-LNA_3_ 3-10-3 gapmer (LNA gapmer A_1_/B_1_ and Scrambled LNA gapmer) and as an LNA_4_-DNA_8_-LNA_4_ 4-8-4 gapmer (LNA gapmer A_2_/B_2_) (Figure 1B).

### 2.2. The LNA Gapmers Bind to the Translation Initiation Region of E. coli rne mRNA

**Figure 2:**
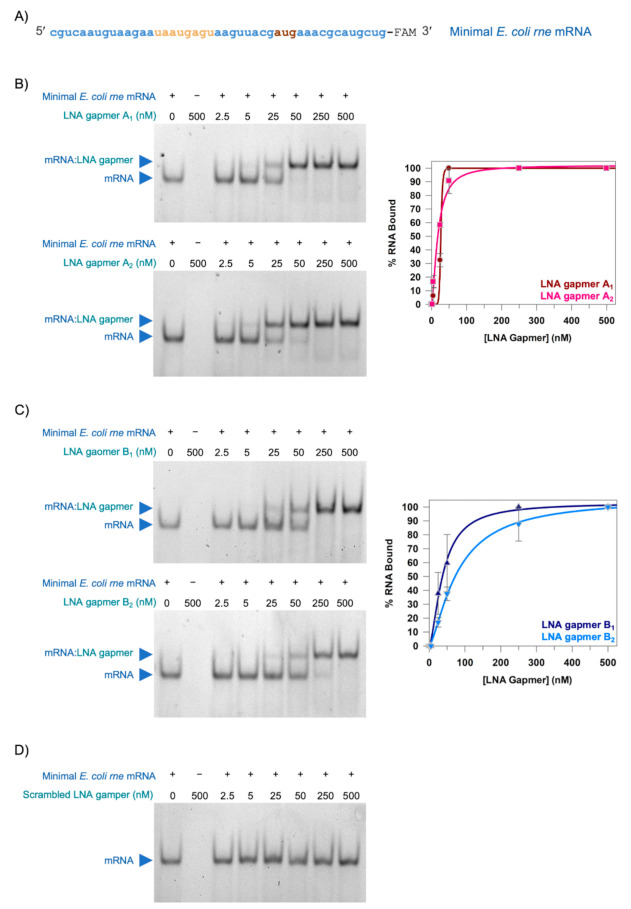
LNA gapmers bind to the translation initiation region of *E. coli rne* mRNA. (**A**) 3′ FAM-labelled 45-mer minimal *E. coli rne* mRNA (blue; RBS, gold; start codon, brown). (**B**–**D**) Representative 12% native-PAGE of minimal *E. coli rne* mRNA incubated with increasing concentrations of LNA gapmer as described in Materials and Methods. (**B**) LNA gapmers A_1_ and A_2_. (**C**) LNA gapmers B_1_ and B_2_. (**D**) Scrambled LNA gapmer. The contrast in the images has been adjusted to aid visualisation. The minimal *E. coli rne* mRNA was present at 50 nM, where indicated (+). The LNA gapmers were present at the indicated concentration. The migration positions of the minimal *E. coli rne* mRNA (mRNA) and the complex of the minimal *E. coli rne* mRNA and the LNA gapmer (mRNA:LNA gapmer) are indicated (blue triangles). Plots of percentage minimal *E. coli rne* mRNA bound against LNA gapmer concentration are shown in (**B**) LNA gapmers A_1_ and A_2_ and (**C**) LNA gapmers B_1_ and B_2_. Data are the mean from three experimental repeats and error bars represent the standard error of the mean (SEM). Data were fit (solid line) to a cooperative binding equation as described in Materials and Methods.

Having designed and synthesised LNA gapmers to target the translation initiation region of *E. coli rne* mRNA, the next step was to determine if they could bind to *E. coli rne* mRNA in vitro. We decided to use an EMSA to evaluate LNA gapmer binding. We designed an unstructured 45-mer minimal *E. coli rne* mRNA corresponding to the −30 to +15 translation initiation region of *E. coli rne* mRNA to use as the target RNA. This region of *E. coli rne* mRNA has been reported to be unstructured in the context of the complete *rne* 5′ UTR [19] and we therefore reasoned that the unstructured minimal *E. coli rne* mRNA would be a suitable RNA target for preliminary experiments. The minimal *E. coli rne* mRNA was synthesised as a 3′ FAM-labelled oligonucleotide (Figure 2A) and incubated with an increasing concentration of each of the LNA gapmers. The reaction mixtures were analysed by native-PAGE (Figure 2B–D).

LNA gapmers A_1_, A_2_, B_1_ and B_2_ all caused an electrophoretic mobility shift of the minimal *E. coli rne* mRNA indicating that they all bound to the minimal *E. coli rne* mRNA to form an mRNA:LNA gapmer complex (Figure 2B,C). In contrast, no mobility shift was observed for minimal *E. coli rne* mRNA in the presence of Scrambled LNA gapmer, even at the highest concentration of LNA gapmer tested (500 nM) indicating that this LNA gapmer did not bind the minimal *E. coli rne* mRNA (Figure 2D). Essentially all of the minimal *E. coli rne* mRNA was present as an mRNA:LNA gapmer complex at LNA gapmer concentrations of 50 nM and above for LNA gapmer A_1_ and LNA gapmer A_2_ (Figure 2B). However, higher LNA gapmer concentrations of 250 nM and above were required for LNA gapmer B_1_ and LNA gapmer B_2_ before all of the minimal *E. coli rne* mRNA was present as an mRNA:LNA gapmer complex (Figure 2C). This suggests that the LNA gapmer A sequence or binding site is preferred over the LNA gapmer B sequence or binding site. In an attempt to quantify this observable difference, the data for LNA gapmers A_1_, A_2_, B_1_ and B_2_ were fit to a cooperative binding equation (Figure 2B,C) and an apparent dissociation constant (K_d_) was calculated for each of these LNA gapmers. The apparent K_d_s for LNA gapmers A_1_ and A_2_ (26.7 ± 4.9 nM and 18.4 ± 2.8 nM, respectively) were lower than the apparent K_d_s for LNA gapmers B_1_ and B_2_ (38.3 ± 3.5 nM and 76.6 ± 7.4 nM, respectively) supporting the qualitative observation that the LNA gapmer A sequence, or binding site, is preferred over the LNA gapmer B sequence, or binding site.

### 2.3. The LNA Gapmers Inhibit Translation in an In Vitro Cell-Free Assay

**Figure 3:**
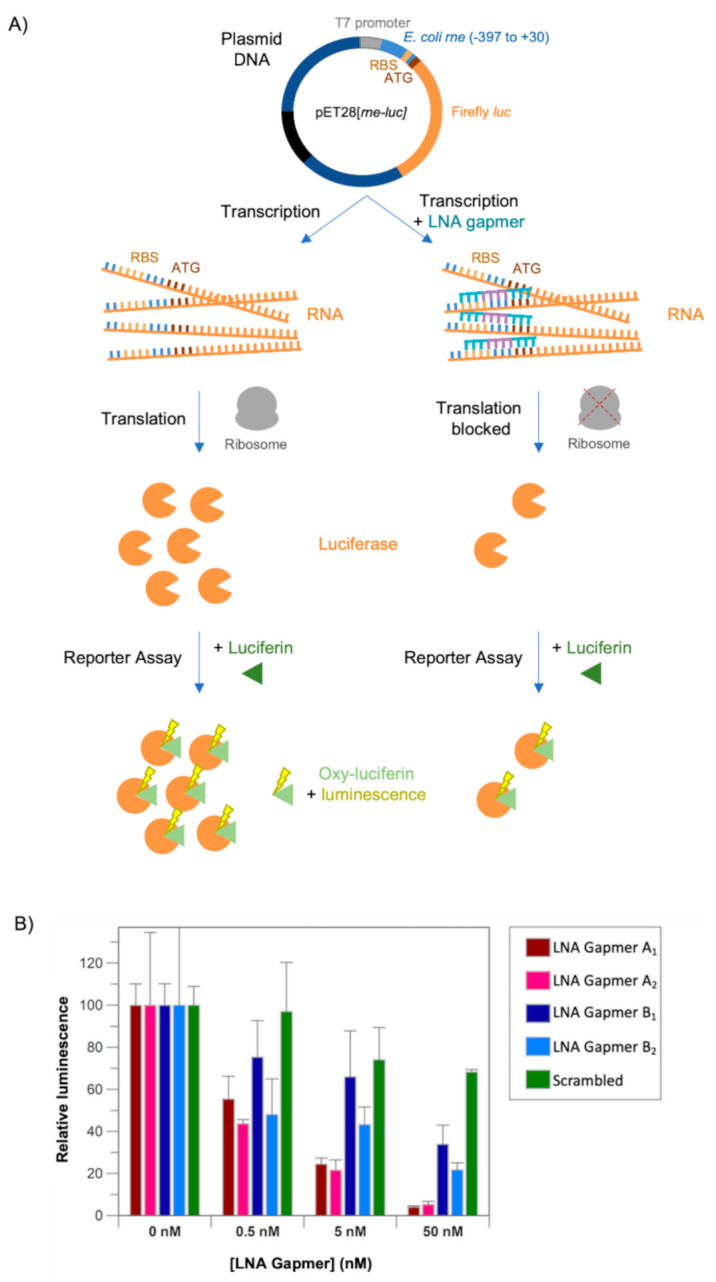
An in vitro cell-free transcription-translation system, coupled with a luciferase assay. (**A**) A schematic of the assay steps. A translational fusion of the −397 to +30 region of *E. coli rne* and the coding region of the firefly luciferase (*luc*) gene (Supplementary Figure S3) was cloned into pET28b to generate pET28[*rne-luc*]. (Left) An in vitro cell-free transcription-translation system transcribes the *rne-luc* gene into RNA (orange; RBS, gold; start codon, brown) and the RNA is translated to produce luciferase (orange wedge). Luciferase converts luciferin (dark green triangle) into oxy-luciferin (light green triangle) and emits light (yellow lightning bolt). (Right) In the presence of LNA gapmer (teal/mauve), translation is inhibited, less luciferase is produced, less luciferin is converted to oxy-luciferin and less light is emitted. (**B**) A chart showing the relative luminescence in the presence of 0 nM, 0.5 nM, 5 nM or 50 nM LNA gapmer A_1_, A_2_, B_1_, B_2_ or Scrambled LNA gapmer. Data have been normalised to the total luminescence observed in the absence of LNA gapmer. Data are the average of three experimental repeats and error bars represent the SEM.

In order to determine whether binding of the LNA gapmers to the translation initiation region of *E. coli rne* mRNA can inhibit translation, an in vitro cell-free transcription-translation system coupled with a luciferase assay was devised. This assay is shown schematically in Figure 3A. A translational fusion of the −397 to +30 region of *rne* and the coding region of the firefly luciferase (*luc*) gene (Supplementary Figure S3) was cloned into pET28b to generate pET28[*rne-luc*]. An in vitro cell-free transcription-translation system was then used to transcribe the *rne-luc* gene and translate it into luciferase. Since luciferase converts luciferin into oxy-luciferin, emitting light in the process, the observed luminescence can provide a readout of the relative level of *rne-luc* mRNA translation or the amount of luciferase present. In the presence of an LNA gapmer, if the LNA gapmer binds to the translation initiation region of *rne* mRNA and inhibits *rne-luc* mRNA translation, it would be expected that less luciferase would be produced and the luminescence would be lower than in the absence of LNA gapmer.

The in vitro cell-free assay was performed in the absence of LNA gapmer and in the presence of 0.5 nM, 5 nM and 50 nM of each of the LNA gapmers (Figure 3B). All of the LNA gapmers, including Scrambled LNA gapmer, which should not bind to *rne-luc* mRNA, negatively affected the total luminescence emitted in a dose-dependent manner. This suggests that the inclusion of any LNA gapmer in the reaction mixture may non-specifically affect *rne-luc* translation and/or luciferase activity. However, at a concentration of 50 nM LNA gapmer, the reduction in luminescence was significantly larger for LNA gapmers A_1_ and A_2_ and LNA gapmers B_1_ and B_2_ than it was for Scrambled LNA gapmer. This is most likely due to the ability of these LNA gapmers to specifically bind to the *rne-luc* mRNA and sterically block its translation. LNA gapmers A_1_ and A_2_ appear to be more potent inhibitors of translation than LNA gapmers B_1_ and B_2_ which is consistent with the higher binding affinity for the translation initiation region of *E. coli rne* mRNA that was observed in the EMSAs (Figure 2B,C). There is also some indication that the 4-8-4 gapmer configuration is more effective than the 3-10-3 gapmer configuration for the LNA gapmer B sequence, which may suggest that the additional LNA nucleotides enhance LNA gapmer stability and/or target binding under these assay conditions.

### 2.4. The LNA Gapmers Stimulate RNase H-Mediated Cleavage of the Translation Initiation Region of E. coli rne mRNA In Vitro

**Figure 4:**
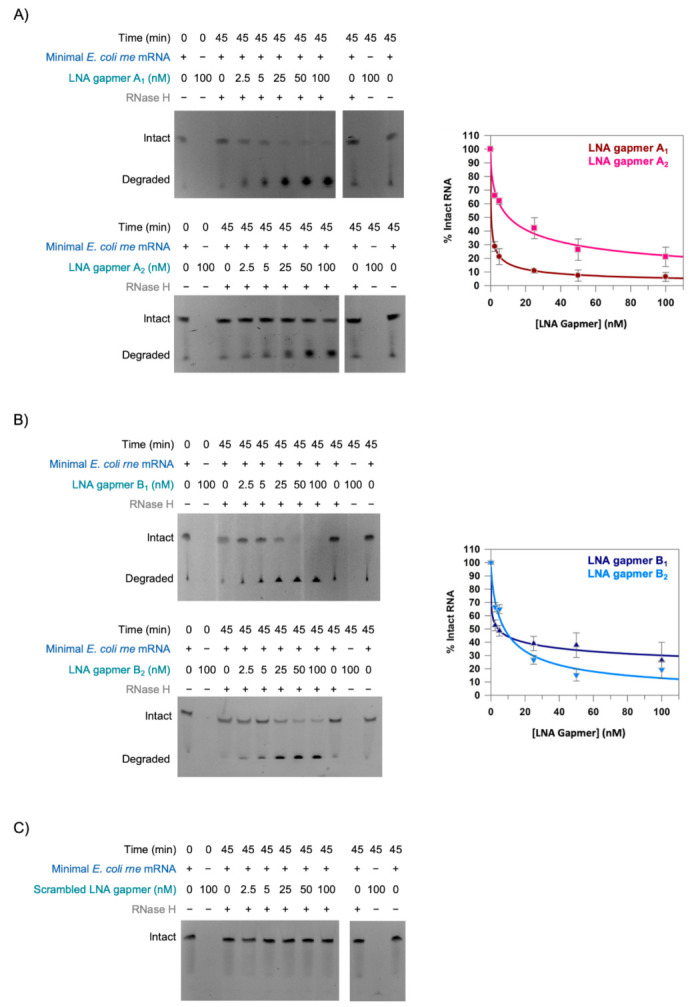
LNA gapmers stimulate RNase H-mediated cleavage of the translation initiation region of *E. coli rne* mRNA. Representative denaturing urea-PAGE analysis of RNase H cleavage assays performed in the presence of increasing concentrations of LNA gapmer as described in Materials and Methods. (**A**) LNA gapmers A_1_ and A_2_. (**B**) LNA gapmers B_1_ and B_2_. (**C**) Scrambled LNA gapmer. The contrast in the images has been adjusted to aid visualisation. The minimal *E. coli rne* mRNA was present at 50 nM, where indicated (+). The LNA gapmers were present at the indicated concentration. RNase H was present at a concentration of 0.008 U/µL, where indicated (+). Note that the cleavage products migrate at or near the visible dye front. For this reason, we focused on the disappearance of the band representing the intact minimal *E. coli rne* mRNA. Plots of the percentage of intact minimal *E. coli rne* mRNA remaining at the end of the RNase H cleavage assay against LNA gapmer concentration are shown in (**A**) LNA gapmers A_1_ and A_2_ and (**B**) LNA gapmers B_1_ and B_2_. Data are the mean from three experimental repeats and error bars represent the SEM. Data were fit (solid line) to a four-parameter logistic function as described in Materials and Methods.

Having determined that the LNA gapmers are capable of binding to the translation initiation region of *E. coli rne* mRNA and inhibiting translation of *rne-luc* mRNA in vitro, presumably by sterically blocking translation, we next wanted to investigate whether they could also recruit RNase H and stimulate RNase H-mediated cleavage of *E. coli rne* mRNA (see Figure 1A for the expected mode-of-action). An in vitro RNase H cleavage assay was developed in which the 3′ FAM-labelled 45-mer minimal *E. coli rne* mRNA (Figure 2A) was used as the target mRNA. The minimal *E. coli rne* mRNA was incubated with an increasing concentration of each of the LNA gapmers in the presence of RNase H and the reaction products were analysed by denaturing urea-PAGE (Figure 4).

As can be seen from the gels in Figure 4, the amount of intact minimal *E. coli rne* mRNA remaining at the end of the assay decreased with increasing concentration of LNA gapmer for LNA gapmers A_1_, A_2_, B_1_ and B_2_ (Figure 4A,B). This suggests that these gapmers all recruit RNase H to the minimal *E. coli rne* mRNA and facilitate RNase H-mediated cleavage of the mRNA. Efficient mRNA cleavage only occurred in the presence of both LNA gapmer and RNase H (Figure 4A,B). In contrast, no cleavage of the minimal *E. coli rne* mRNA was observed in the presence of Scrambled LNA gapmer, even at the highest LNA gapmer concentration (100 nM) tested (Figure 4C).

In order to try to quantitate the effect of the different LNA gapmers on RNase H recruitment and RNase H-mediated mRNA cleavage, the data for LNA gapmers A_1_, A_2_, B_1_ and B_2_ were fit to a four-parameter logistic function (Figure 4A,B) which allowed us to estimate the half maximal inhibitory concentration (IC_50_) for each of them. When referring to this as inhibition, we considered cleavage of the minimal *E. coli rne* mRNA to represent inhibition of mRNA function. This analysis suggested that LNA gapmer A_1_, with an IC_50_ of 0.4 ± 0.1 nM, was the most effective of all of the LNA gapmers to recruit RNase H and stimulate RNase H-mediated cleavage of the minimal *E. coli rne* mRNA. The IC_50_ for LNA gapmer A_2_, at 10.5 ± 2.8 nM, was approximately 25-fold higher than for LNA gapmer A_1_. Since LNA gapmers A_1_ and A_2_ have the same nucleotide sequence, this implies that the difference is a consequence of the 3-10-3 LNA gapmer configuration of LNA gapmer A_1_ compared to the 4-10-4 LNA gapmer configuration of LNA gapmer A_2_. However, this trend between the 3-10-3 and 4-8-4 LNA gapmer configurations was less apparent for the LNA gapmer B sequence with IC_50_s of 4.2 ± 1.9 nM for the 3-10-3 LNA gapmer B_1_ and 8.0 ± 2.9 nM for the 4-8-4 LNA gapmer B_2_.

## 3. Discussion

Increasing antibacterial resistance has led to a need for novel antibacterial targets and novel antibacterial strategies. RNase E/*rne* has been identified as a prospective antibacterial target [3,4] while antisense oligonucleotides hold potential as an antibacterial strategy [14,15,16,17]. In this study we combined both of these novel factors to target *rne* mRNA with antisense oligonucleotides. Specifically, we have successfully designed two antisense oligonucleotide sequences to target *E. coli rne* mRNA and have demonstrated that they have the requisite translation blocking activity and the ability to recruit RNase H and facilitate mRNA cleavage in vitro. This work provides the foundation for the development of an antibacterial strategy targeting RNase/*rne*.

The earliest antisense oligonucleotides targeted viral RNAs [21,22]. This pioneering work highlighted the potential of antisense approaches to rationally target essentially any RNA to combat a variety of infections and/or diseases. Although there has been significant progress in the development of therapeutic antisense oligonucleotides to target disease, such as neurodegenerative disorders, cardiovascular disorders and cancer (reviewed in [23,24,25]), antisense oligonucleotides for antibacterial applications are yet to make it to the clinic. One of the main reasons why antibacterial antisense oligonucleotide research lags behind other RNA therapeutics is the challenge of cellular uptake [17]. There has been far less focus on antisense oligonucleotide delivery to bacterial cells than to eukaryotic cells, and the advances that have been made in the eukaryotic field are not broadly applicable to prokaryotic systems [17]. Due to the known challenges with antisense oligonucleotide delivery to bacterial cells, it is best practice to investigate the plausibility of targeting a bacterial mRNA target with antisense oligonucleotides in vitro in the first instance [17], just as we have done here for *E. coli rne* mRNA.

Indeed, our results demonstrate that it is feasible to target *E. coli rne* mRNA with antisense oligonucleotides. In addition, our comparison of different antisense oligonucleotide sequences, different LNA gapmer configurations and different modes-of-action of the LNA gapmers has provided some key knowledge to take forwards into in vivo studies. As might have been expected, the LNA gapmer A sequence which targets both the RBS and start codon of the *E. coli rne* mRNA translation initiation region was more efficient at inhibiting translation than the LNA gapmer B sequence which only targets the start codon (Figure 3). It is possible that preventing ribosome binding may be a better strategy than blocking ribosome progression. However, this result may simply reflect the different binding affinities observed for the LNA gapmer A and B sequences (Figure 2). The origin of these different binding affinities is unclear. It is known that the structural context of the antisense oligonucleotide binding site is a critical determinant for its binding affinity [26]. However, translation initiation regions of mRNAs are typically unstructured to allow access to ribosomes [17] and the *E. coli rne* mRNA translation initiation region has been reported to be unstructured [19]. Therefore, there is no obvious difference in the structural context of the binding site for LNA gapmer A compared to LNA gapmer B.

The comparison of the 3-10-3 and 4-8-4 LNA gapmer configurations generated mixed results. There were hints that the 4-8-4 LNA gapmer configuration performed better with regard to inhibiting translation (Figure 3). This might be explained by the increased LNA content stabilising the mRNA:LNA gapmer complex. In contrast, the 3-10-3 gapmer configuration appeared to perform better with regard to RNase H recruitment and mRNA cleavage (Figure 4). This might be explained if either the higher DNA content of 3-10-3 LNA gapmers enhances RNase H recruitment, or the lower binding affinity of the 3-10-3 LNA gapmers leads to higher LNA gapmer recycling, relative to the 4-8-4 LNA gapmers [27].

Interestingly, although there may be preferred sequences and/or LNA gapmer configurations, our findings suggest that antisense oligonucleotides can efficiently down-regulate *rne* mRNA expression by either sterically blocking translation or stimulating RNase H-mediated mRNA cleavage. The majority of antisense oligonucleotides that have been reported to have antibacterial activity are either entirely PMO or entirely PNA (Supplementary Table S1), are not recognised by RNase H, and are therefore restricted to down-regulating gene expression by sterically blocking translation. This is in contrast to wider RNA therapeutic applications where the value of combining oligonucleotide chemistries, e.g., as gapmers, to optimise antisense oligonucleotide properties and mode-of-action is now recognised [17,18,23]. Incompatible assay conditions meant that, unfortunately, we were unable to test the effect of combining the two modes of action and it remains to be seen whether there is a preferred mode-of-action in vivo.

Having demonstrated the successful targeting of *E. coli rne* mRNA with antisense oligonucleotides in vitro, the next step would be to investigate their activity, particularly in regard to their antibacterial properties, in vivo. As discussed above, delivery of antisense oligonucleotides to bacterial cells is challenging [17] and a “naked” LNA gapmer would not be expected to enter bacterial cells. Not surprisingly, preliminary Kirby-Bauer disk diffusion assays [28] with LNA gapmers A_1_ and A_2_, and *E. coli*, showed no inhibition of bacterial growth. The most common strategy for facilitating antisense oligonucleotide delivery is the conjugation of a cell-penetrating peptide (CPP), e.g., (KFF)_3_F [17,29,30,31,32,33], to the antisense oligonucleotide. This strategy has been used to deliver PNA antisense oligonucleotides into *E. coli* [29] and LNA antisense oligonucleotides into *Staphylococcus aureus* [32] and would, therefore, be a good place to start for the *E. coli rne* mRNA-targeting LNA gapmers. Alternative strategies, such as the use of nanomaterials, are rarely used for antibacterial antisense oligonucleotides [17]. Although, progress is being made in terms of using cationic vesicles (bolasomes) to deliver antisense oligonucleotides into *Clostridium difficile* [34]. Cellular uptake could be confirmed by using a fluorescently labelled CPP-LNA gapmer and confocal microscopy [17].

**Figure 5:**
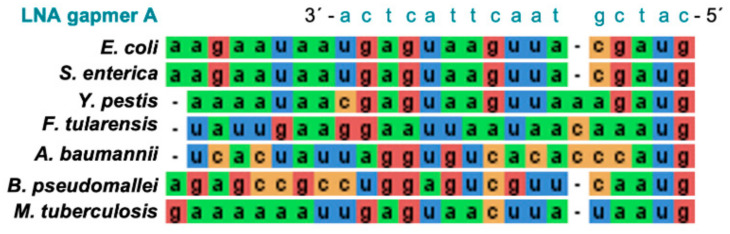
Sequence alignment of the −21 to +3 translation initiation region of *rne* mRNA. A sequence alignment of the −21 to +3 translation initiation region of the *rne* mRNA from *Escherichia coli*, *Salmonella enterica, Yersinia pestis, Francisella tularensis, Acinetobacter baumannii, Burkolderia pseudomallei* and *Mycobacterium tuberculosis*. Sequences were aligned using MAFFT [35] and coloured by nucleotide in JalView [36]. The complementary LNA gapmer A sequence is shown above the alignment to indicate the LNA gapmer A_1_/A_2_ binding site.

It would also be interesting to investigate whether the antisense oligonucleotides designed here to target *E. coli rne* mRNA could be effective against *rne* mRNA from other bacterial species. Small molecule inhibitors of *E. coli* RNase E also inhibited RNase E from other bacteria suggesting potential as lead compounds in the development of broad spectrum antibiotics [12,13]. As shown in Figure 5, the region where the LNA gapmers A_1_ and A_2_ bind to *E. coli rne* mRNA, is absolutely conserved in the *rne* mRNA of the closely related bacterium *S. enterica*. However, in another closely related bacterium, *Y. pestis*, there is sequence variation. This sequence variation becomes more pronounced in more distantly related bacteria such as *Francisella tularensis, Acinetobacter baumannii* and *Burkolderia pseudomallei*. In contrast, the *rne* translation region in *Mycobacteriun tuberculosis* is similar to that in *E. coli*. Experimental validation will be needed to ascertain how much sequence variation can be tolerated before an LNA gapmer fails to have an effect. This will be important for tailoring antibacterial strategies and also for combatting the emergence of antibacterial resistant mutants.

In summary, we have successfully designed two novel antisense oligonucleotide sequences to target *E. coli rne* mRNA, a novel antibacterial target. We synthesised four LNA gapmers based on these sequences and demonstrated that they bind to *E. coli rne* mRNA, inhibit translation of *E. coli rne* mRNA and facilitate RNase H recruitment and mRNA cleavage in vitro. Given these activities, it is anticipated that these LNA gapmers will display antibacterial activity in vivo. Although the challenge of antisense oligonucleotide delivery remains, there are feasible strategies available. Therefore, this work provides the foundation for a possible novel antibacterial strategy targeting *rne* mRNA.

## 4. Materials and Methods

### 4.1. LNA Gapmer Design and Synthesis

Two 16-mer antisense oligonucleotide sequences (sequence A: 5′ CATCGTAACTTACTCA 3′; sequence B: 5′ GCGTTTCATCGTAACT 3′) were designed to target the −30 to +15 translation initiation region of the *E. coli rne* gene (5′ CGUCAAUGUAAGAAUAAUGAGUAAGUUACGAUGAAACGCAUGCUG 3′). Each sequence was synthesised as an LNA_3_-DNA_10_-LNA_3_ 3-10-3 gapmer (LNA gapmer A_1_/B_1_) and as an LNA_4_-DNA_8_-LNA_4_ 4-8-4 gapmer (LNA gapmer A_2_/B_2_). The oligonucleotides were synthesised under standard conditions at 1 µmol scale using an ABI 394 DNA Synthesizer (Biolytic Lab Performance, Fremont, CA, USA) on 1000 Å UnyLinker-functionalised LCAA CPG support. The oligonucleotides were subsequently cleaved from support and the Unylinker moiety and protecting groups were removed by treatment with concentrated aqueous ammonia at 55 °C overnight. The solution was decanted and dried using a centrifugal evaporator, then the pellets were diluted in 1 mL milliQ water and purified using ion exchange HPLC. The oligonucleotides were characterised using LC-MS (ESI-mode) and the concentration of the final solutions was determined according to their absorbance at 260 nm.

A scrambled 16-mer sequence (5′ ATCTACCAAATTTCCG 3′) was also generated based on sequence A using Shuffle DNA [37]. This sequence was synthesised at 0.05 µmole scale as an LNA_3_-DNA_10_-LNA_3_ 3-10-3 gapmer (Scrambled LNA gapmer) and HPLC-purified by Merck (Merck Life Science UK Limited, Gillingham, UK).

### 4.2. Design and Synthesis of a Minimal E. coli rne mRNA

A 3′ 6-fluorescein amidate (FAM)-labelled RNA oligonucleotide corresponding to the −30 to +15 translation initiation region of the *E. coli rne* gene (RNA; 5′ CGUCAAUGUAAGAAUAAUGAGUAAGUUACGAUGAAACGCAUGCUG-[FAM] 3′) was synthesised at 1 µmole scale and HPLC-purified by Sigma-Aldrich (now Merck Life Science UK Limited). The concentration of FAM-labelled RNA was determined according to the absorbance at 260 nm using a conversion factor of 0.26 to correct for the 6-FAM absorbance [38]. Denaturing urea-PAGE was used to confirm that the RNA was a single species of the expected size.

### 4.3. Electrophoretic Mobility Shift Assays (EMSAs)

A 10× (500 nM) stock of minimal *E. coli rne* mRNA was prepared in EMSA reaction buffer (10 mM Tris-HCl (pH 8.0), 50 mM NaCl, 50 mM KCl, 0.5 mM ethylenediaminetertraacetic acid (EDTA), 10% glycerol), heated at 80 °C for 10 min, cooled at room temperature for 10 min and then equilibrated at 37 °C for 10 min. 10× stocks of each of the LNA gapmers were prepared at concentrations of 25 nM, 50 nM, 250 nM, 500 nM, 2.5 µM and 5 µM in EMSA reaction buffer and equilibrated at 37 °C for 10 min. 10 µL reaction mixtures containing 50 nM minimal *E. coli rne* mRNA and 0 nM, 2.5 nM, 5 nM, 25 nM, 50 nM, 250 nM or 500 nM LNA gapmer in EMSA reaction buffer were assembled and incubated at room temperature for 10 min. Reactions were analysed by 12% native-PAGE run in Tris-borate-EDTA (TBE) running buffer at 80 V for 2 h at room temperature. Gels were visualised using a GBox UV transilluminator (Syngene, a division of Synoptics Ltd., Cambridge, UK). Digitised images were quantitated using ImageJ (Rasband, W.S., ImageJ, U.S. National Institutes of Health, Bethesda, MD, USA, https://imagej.nih.gov/ij/, 1997–2018) and the percentage of bound and unbound RNA in each lane was calculated. Data from triplicate experiments were fit in Grafit5 (Erithacus Software, Grinstead, West Sussex, UK) to a cooperative binding equation:

(1) $$y=\frac{L^{n}.Cap}{K^{n}+{\left[L\right]}^{n}}+background$$

In this equation, *y* is the percentage of FAM-labelled RNA bound by LNA gapmer, [*L*] is the concentration of LNA gapmer, *n* is the slope factor, *Cap* is the theoretical maximal amount of FAM-labelled RNA than can be bound by LNA gapmer, *K* is the apparent equilibrium dissociation constant (also termed apparent K_d_) and *background* allows for any *y*-axis displacement from the origin.

### 4.4. Cell-Free Reporter Assay

#### 4.4.1. Design and Synthesis of the *E. coli rne*-Firefly Luciferase (*luc*) Reporter Plasmid

A translational fusion of the −397 to +30 region of *E. coli rne* and the coding region of the firefly luciferase (*luc*) gene (Supplementary Figure S3) was synthesised by GeneArt (Thermo Fisher Scientific, Waltham, MA, USA) and ligated between the *XbaI* and *XhoI* restriction sites of pET28b (Novagen, a brand of Merck, Darmstadt, Germany) to generate pET28[*rne*-*luc*]. The sequence was confirmed by DNA sequencing. *E. coli* DH5α was transformed with pET28[*rne*-*luc*]. DH5α, pET28[*rne*-*luc*] was grown in LB supplemented with 25 µg/mL kanamycin at 37 °C overnight with shaking. Cells were harvested by centrifugation and pET28[*rne*-*luc*] was extracted using the NucleoBond Xtra Midi plasmid preparation kit (Macherey-Nagel, Düren, Germany).

#### 4.4.2. In Vitro Transcription-Translation Real-Time Reporter Assay

In vitro transcription-translation of pET28 [*rne-luc*] was performed using the *E. coli* T7 S30 Extract System for Circular DNA (Promega, Madison, WI, USA). 50 µL reactions were prepared in a 96-well plate. Each reaction contained 750 ng pET28 [*rne-luc*], 1 mM luciferin (BD Biosciences, BD Biosciences, San Jose, CA, USA), 5 µL Complete Amino Acid Mixture, 20 µL S30 Premix and 15 µL T7 S30 Extract. The reactions were supplemented with 0.5 nM, 5 nM or 50 nM LNA gapmer, as indicated. The reactions were not supplemented with exogenous RNase H. Reactions were incubated at 37 °C for 2 h in a Hidex Sense plate-reader (Hidex Ltd., Turku, Finland). The luminescence (or luciferase signal) was recorded every 2.5 min for a total of 120 min. The total luciferase signal for each LNA gapmer concentration (0 nM, 0.5 nM, 5 nM and 50 nM) was quantitated by integrating the area under the curve using a trapezoid method in Excel. Integrated values were normalised to a percentage (relative to the total luciferase signal in the absence of LNA gapmer) and plotted as a bar chart in Grafit5.

### 4.5. RNase H Cleavage Assay

Twenty five µL reaction mixtures containing 50 nM minimal *E. coli rne* mRNA and 0 nM, 2.5 nM, 5 nM, 25 nM, 50 nM or 100 nM LNA gapmer in 1× RNase H Reaction Buffer (NEB, Ipswich, MA, USA; 75 mM KCl, 50 mM Tris-HCl pH 8.3, 3 mM MgCl_2_, 10 mM DTT) were assembled and incubated at 37 °C for 5 min to allow LNA gapmer to bind to the target minimal *E. coli rne* mRNA and form the RNase H substrate. RNase H, equilibrated at 37 °C, was then added to a final concentration of 0.008 U/µL. The complete reaction mixture was incubated at 37 °C for a further 45 min. Reactions were terminated by the addition of 0.5 volumes of quench buffer (95% (*v*/*v*) formamide, 18 mM EDTA). Reaction mixtures were heated at 95 °C for 5 min and reaction products were resolved by 8% denaturing urea-PAGE. Gels were visualised using a GBox UV transilluminator (Syngene, a division of Synoptics Ltd., Cambridge, UK). Digitised images were quantitated using ImageJ and the percentage of cleaved and uncleaved FAM-labelled RNA in each lane was calculated. Data from triplicate experiments were fit to a four-parameter logistic function to estimate the half maximal inhibitory concentration (IC_50_):

(2) $$y=\frac{Range}{1+{\left(\frac{x}{IC_{50}}\right)}^{s}}+background$$

In this equation, *y* is the percentage of intact FAM-labelled RNA at LNA gapmer concentration *x*; *Range* is the theoretical extent of the reaction, *IC*_50_ is the concentration of LNA gapmer at half the *Range*, *s* is the slope factor and *background* allows for any *y*-axis displacement from the origin.

## Data Availability

The data presented in this study are available on request from the corresponding authors.

## Supplementary Materials

The following are available online, Supplementary References, Supplementary Table S1: Examples of antibacterial antisense oligonucleotides, Supplementary Figure S1: Mode-of-action of antisense oligonucleotides, Supplementary Figure S2: Unmodified nucleic acids and examples of chemical analogues used in antisense oligonucleotides, Supplementary Figure S3: The *E. coli rne*-firefly luciferase reporter.

## Sample Availability

Not available.
