# Supplementary material for: An Investigation into the Potential of Targeting Escherichia coli rne mRNA with Locked Nucleic Acid (LNA) Gapmers as an Antibacterial Strategy
Source: Molecules. 2021 Jun 4;26(11):3414. doi: 10.3390/molecules26113414 (PMC8200214; doi:10.3390/molecules26113414)
Supplement: Supplementary file 1 [file molecules-26-03414-s001.zip › molecules-1179771-supplementary.pdf]

Supplementary Material

An investigation into the potential of targeting *Escherichia coli rne* mRNA with locked nucleic acid (LNA) gapmers as an antibacterial strategy

Layla R. Goddard[1,2,‡], Charlotte E. Mardle[1,‡], Hassan Gneid,[3,4,5], Ciara G. Ball[1,2], Darren M. Gowers[1], Helen S. Atkins[6,7,8], Louise E. Butt[1], Jonathan K. Watts[3], Helen A. Vincent[1,2,*] and Anastasia J. Callaghan[1,2,*]

[1] School of Biological Sciences and Institute of Biological & Biomedical Sciences, University of Portsmouth, Portsmouth, PO1 2DY, United Kingdom

[2] Centre for Enzyme Innovation, University of Portsmouth, Portsmouth, PO1 2DY, United Kingdom

[3] RNA Therapeutics Institute, University of Massachusetts Medical School, Worcester, MA, 01609, USA.

[4] Department of Chemistry, University of Southampton, Southampton, SO17 1BJ, UK.

[5] Current address: Department of Chemistry, Tulane University, 6823 St. Charles Avenue, New Orleans, LA 70118

[6] Defence Science and Technology Laboratory, Porton Down, Salisbury, United Kingdom

[7] University of Exeter, Exeter, United Kingdom

[8] London School of Hygiene and Tropical Medicine, London, United Kingdom

‡ These authors contributed equally to this work.

* Correspondence: Anastasia.Callaghan@port.ac.uk (A.J.C.), Helen.Vincent@port.ac.uk (H.A.V.)

Supplementary References

1. Rapaport, E., Levina, A., Metelev, V. and Zamecnik, P. C. (1996) Antimycobacterial activities of antisense oligodeoxynucleotide phosphorothioates in drug-resistant strains. *Proc. Natl. Acad. Sci. U. S. A.* **93**, 709-713. doi: 10.1073/pnas.93.2.709.

2. Harth, G., Zamecnik, P. C., Tang, J. Y., Tabatadze, D. and Horwitz, M. A. (2000) Treatment of *Mycobacterium tuberculosis* with antisense oligonucleotides to glutamine synthetase mRNA inhibits glutamine synthetase activity, formation of the poly-L-glutamate/glutamine cell wall structure, and bacterial replication. *Proc. Natl. Acad. Sci. USA* **97**, 418-423. doi: 10.1073/pnas.97.1.418.

3. Good, L., Awasthi, S. K., Dryselius, R., Larsson, O. and Nielsen, P. E. (2001) Bactericidal antisense effects of peptide-PNA conjugates. *Nat. Biotechnol.* **19**, 360-364. doi: 10.1038/86753.

4. Tan, X. X., Actor, J. K. and Chen, Y. (2005) Peptide nucleic acid antisense oligomer as a therapeutic strategy against bacterial infection: proof of principle using mouse intraperitoneal infection. *Antimicrob. Agents Chemother.* **49**, 3203-3207. doi: 10.1128/AAC.49.8.3203-3207.2005.

5. Nikravesh, A., Dryselius, R., Faridani, O. R., Goh, S., Sadeghizadeh, M., Behmanesh, M., Ganyu, A., Klok, E. J., Zain, R. and Good, L. (2007). Antisense PNA accumulates in *Escherichia coli* and mediates a long post-antibiotic effect. *Mol. Ther.* **15**, 1537–1542. doi: 10.1038/sj.mt.6300209.

6. Geller, B. L., Deere, J. D., Stein, D. A., Kroeker, A. D., Moulton, H. M. and Iversen, P. L. (2003) Inhibition of gene expression in *Escherichia coli* by antisense phosphorodiamidate morpholino oligomers. *Antimicrob. Agents Chemother.* **47**, 3233-3239. doi: 10.1128/aac.47.10.3233-3239.2003.

7. Deere, J., Iversen, P. and Geller, B. L. (2005) Antisense phosphorodiamidate morpholino oligomer length and target position effect on gene-specific inhibition in *Escherichia coli*. *Antimicrob. Agents Chemother.* **49**, 249-255. doi: 10.1128/AAC.49.1.249-255.2005.

8. Geller, B. L., Deere, J., Tilley, L. and Iversen, P. L. (2005) Antisense phosphorodiamidate morpholino oligomer inhibits viability of *Escherichia coli* in pure culture and in mouse peritonitis. *J. Antimicrob. Chemother.* **55**, 983-988. doi: 10.1093/jac/dki129.

9. Tilley, L. D., Mellbye, B. L., Puckett, S. E., Iversen, P. L. and Geller, B. L. (2007) Antisense peptide-phosphorodiamidate morpholino oligomer conjugate: dose-response in mice infected with *Escherichia coli*. *J. Antimicrob. Chemother.* **59**, 66-73. doi: 10.1093/jac/dkl444.

10. Mellbye, B. L., Puckett, S. E., Tilley, L. D., Iversen, P. L. and Geller, B. L. (2009) Variation in amino acid composition of antisense peptide-phosphorodiamidate morpholino oligomer affect potency against *Escherichia coli in vitro* and *in vivo*. *Antimicrob. Agents Chemother.* **53**, 525-530. doi: 10.1128/AAC.00917-08.

11. Mellbye, B. L., Weller, D. D., Hassinger, J. N., Reeves, M. D., Lovejoy, C. E., Iversen, P. L. and Geller, B. L. (2010) Cationic phosphorodiamidate morpholino oligomers efficiently prevent growth of *Escherichia coli in vitro* and *in vivo*. *J. Antimicrob. Chemother.* **65**, 98-106. doi: 10.1093/jac/dkp392.

12. Mitev, G. M., Mellbye, B. L., Iversen, P. L. and Geller, B. L. (2009) Inhibition of intracellular growth of *Salmonella enterica* serovar Typhimurium in tissue culture by antisense peptide-phosphorodiamidate morpholino oligomer. *Antimicrob. Agents Chemother.* **53**, 3700-3704. doi: 10.1128/AAC.00099-09.

13. Greenberg, D. E., Marshall-Batty, K. R., Brinster, L. R., Zarember, K. A., Shaw, P. A., Mellbye, B. L., Iversen, P. L., Holland, S. M. and Geller, B. L. (2010) Antisense phosphorodiamidate morpholino oligomers targeted to an essential gene inhibit *Burkholderia cepacia* complex. *J. Infect. Dis.* **201**, 1822-1830. doi: 10.1086/652807.

14. Panchal, R. G., Geller, B. L., Mellbye, B., Lane, D., Iversen, P. L. and Bavari, S. (2012) Peptide conjugated phosphorodiamidate morpholino oligomers increase survival of mice challenged with Ames *Bacillus anthracis*. *Nucleic Acid. Ther.* **22**, 316-322. doi: 10.1089/nat.2012.0362.

15. Ghosal, A. and Nielsen, P. E. (2012) Potent antibacterial antisense peptide-peptide nucleic acid conjugates against *Pseudomonas aeruginosa*. *Nucleic Acid. Ther.* **22**, 323-334. doi: 10.1089/nat.2012.0370.

16. Howard, J. J., Sturge, C. R., Moustafa, D. A., Daly, S. M., Marshall-Batty, K. R., Felder, C. F., Zamora, D., Yabe-Gille, M., Labandeira-Rey, M., Bailey, S., M., Wong, M., Goldberg, J. B., Geller, B. L. and Greenberg, D. E. (2017) Inhibition of *Pseudomonas aeruginosa* by peptide-conjugated phosphorodiamidate morpholino oligomers. *Antimicrob. Agents Chemother.* **61**, e01938-16. doi: 10.1128/AAC.01938-16.

17. Geller, B. L., Marshall-Batty, K., Schnell, F. J., McKnight, M. M., Iversen, P. L. and Greenberg, D. E. (2013) Gene-silencing antisense oligomers inhibit *acinetobacter* growth *in vitro* and *in vivo*. **208**, 1553-1560. doi: 10.1093/infdis/jit460.

18. Otsuka, T., Brauer, A. L., Kirkham, C., Sully, E. K., Pettigrew, M. M., Kong, Y, Geller, B. L. and Murphy, T. F. (2017) Antimicrobial activity of antisense peptide-peptide nucleic acid conjugates against non-typeable *Haemophilus influenzae* in planktonic and biofilm forms. *J. Antimicrob. Chemother.* **72**, 137-144. doi: 10.1093/jac/dkw384.

19. Nekhotiaeva, N., Awasthi, S. K., Nielsen, P. E. and Good, L. (2004) Inhibition of *Staphylococcus aureus* gene expression and growth using antisense peptide nucleic acids. *Mol. Ther.* **10**, 652-659. doi: 10.1016/j.ymthe.2004.07.006.

20. Kurupati, P., Tan, K. S., Kumarasinghe, G. and Poh, C. L. (2007) Inhibition of gene expression and growth by antisense peptide nucleic acids in a multiresistant beta-lactamase-producing *Klebsiella pneumoniae* strain. *Antimicrob. Agents Chemother.* **51**, 805-811. doi: 10.1128/AAC.00709-06.

21. Wesolowski, D., Alonso, D. and Altman, S. (2013) Combined effect of a peptide-morpholino oligonucleotide conjugate and a cell-penetrating peptide as an antibiotic. *Proc. Natl. Acad. Sci. U. S. A.* **110**, 8686-8689. doi: 10.1073/pnas.1306911110.

22. Patenge, N., Pappesch, R., Krawack, F., Walda, C., Mraheil, M. A., Jacob, A., Hain, T. and Kreikemeyer, B. (2013) Inhibition of growth and gene expression by PNA-peptide conjugates in *Streptococcus pyogenes*. *Mol. Ther. Nucleic Acids* **2**, e132. doi: 10.1038/mtna.2013.62.

23. Kulyté, A., Nekhotiaeva, N. Awasthi, S. K. and Good, L. (2005) Inhibition of *Mycobacterium smegmatis* gene expression and growth using antisense peptide nucleic acids. *J. Mol. Microbiol. Biotechnol.* **9**, 101-109. doi: 10.1159/000088840.

24. Li, Y., Chen, Z., Li, X., Zhang, H., Huang, Q., Zhang, Y. and Xu, S. (2007) Inositol-1-phosphate synthetase mRNA as a new target for antisense inhibition of *Mycobacterium tuberculosis*. *J. Biotechnol.* **128**, 726-734. doi: 10.1016/j.jbiotec.2006.12.019.

25. Bai, H., You, Y., Yan, H., Meng, J., Xue, X., Hou, Z., Zhou, Y., Ma, X., Sang, G. and Luo, X. (2012) Antisense inhibition of gene expression and growth in Gram-negative bacteria by cell-penetrating peptide conjugates of peptide nucleic acids target to *rpoD* gene. *Biomaterials* **33**, 659-667. doi: 10.1016/j.biomaterials.2011.09.075.

26. Alajlouni, R. A. and Seleem, M. N. (2013) Targeting *Listeria monocytogenes rpoA* and *rpoD* genes using peptide nucleic acids. *Nucleic Acid Ther.* **23**, 363-367. doi: 10.1089/nat.2013.0426.

27. Meng, J., Da, F., Ma, X., Wang, N., Wang, Y., Zhang, H., Li, M., Zhou, Y., Xue, X., Hou, Z., Jia, M. and Luo, X. (2015) Antisense growth inhibition of methicillin-resistant *Staphylococcus aureus* by locked nucleic acid conjugated with cell-penetrating as a novel FtsZ inhibitor. *Antimicrob. Agents Chemother.* **59**, 914-922. doi: 10.1128/AAC.03781-14.

28. Liang, S., He, Y., Xia, Y., Wang, H., Wang, L., Gao, R. and Zhang, M. (2015) Inhibiting the growth of methicillin resistant *Staphylococcus aureus in vitro* with antisense peptide nucleic acid conjugates targeting the *ftsZ* gene. *Int. J. Infect. Dis.* **30**, 1-6. doi: 10.1016/j.ijid.2014.09.015.

29. Da, F., Yao, L., Su, Z., Hou, Z., Li, Z., Xue, X., ... & Luo, X. (2017). Antisense locked nucleic acids targeting *agrA* inhibit quorum sensing and pathogenesis of community-associated methicillin-resistant *Staphylococcus aureus*. *J. Appl. Microbiol.* **122**, 257-267. doi: 10.1111/jam.13321.

30. Rose, M., Lapuebla, A., Landman, D. and Quale, J. (2019) *In vitro* and *In vivo* activity of a novel antisense peptide nucleic acid compound against multidrug-resistant *Acinetobacter baumannii*. *Microb. Drug Resist.* **25**, 961-965. doi: 10.1089/mdr.2018.0179.

31. Khvorova, A. and Watts, J. K. (2017) The chemical evolution of oligonucleotide therapies of clinical utility. *Nat. Biotech.* **35**, 238-248. doi: 10.1038/nbt.3765.

Supplementary Table S1. Examples of antibacterial antisense oligonucleotides.

Genetic target	Bacterial target	Chemistry of antisense oligonucleotide	Reference(s)
ask (aspartokinase; amino acid biosynthesis)	*Mycobacterium smegmatis*	Phosphorothioate	[1]
*gln*A1 (glutamine synthetase; nitrogen metabolism)	*Mycobacterium tuberculosis*	Phosphorothioate	[2]
acpP (acyl carrier protein; fatty acid biosynthesis)	*Escherichia coli*	Peptide-peptide nucleic acid (PNA) conjugate	[3-5]
		PNA	[4]
		Phosphorodiamidate morpholino (PMO)	[6-8]
		Peptide-PMO conjugate	[9-11]
	Salmonella enterica	Peptide-PMO conjugate	[12]
	Burkholderia cepacia	Peptide-PMO conjugate	[13]
	Bacillus anthracis	Peptide-PMO conjugate	[14]
	Psuedomonas aeruginosa	Peptide-PMO conjugate	[15,16]
	Acinetobacter lwoffii	Peptide-PMO conjugate	[17]
	Acinetobacter baumannii	Peptide-PMO conjugate	[17]
	Haemophilus influenzae	Peptide-PNA conjugate	[18]
gyrA (DNA gyrase; DNA replication)	*Staphylococcus aureus*	Peptide-PNA conjugate	[19]
	Klebsiella pneumoniae	Peptide-PNA conjugate	[20,21]
	Bacillus anthracis	Peptide-PMO conjugate	[14]
	E. coli	Peptide-PMO conjugate	[21]
	M. smegmatis	Peptide-PMO conjugate	[21]
	A. baumannii	Peptide-PMO conjugate	[21]
	Bacillus subtilis	Peptide-PMO conjugate	[21]
	Salmonella typhimurium	Peptide-PMO conjugate	[21]
	Streptococcus pyogenes	Peptide-PNA conjugate	[22]
fmhB (peptidoglycan biosynthesis)	*S. aureus*	Peptide-PNA conjugate	[19]
hmrB (acyl carrier protein; fatty acid biosynthesis)	*S. aureus*	Peptide-PNA conjugate	[19]
inhA (mycolic acid biosynthesis)	*M. smegmatis*	Peptide-PNA conjugate	[23]
ompA (outer membrane protein; porin)	*K. pneumoniae*	Peptide-PNA conjugate	[20]
ino1 (inositol-3-phosphate synthase	*M. tuberculosis*	Phosphorothioate	[24]
rpoD (RNA polymerase sigma factor; transcription)	*E. coli*	Peptide-PNA conjugate	[25]
	S. enterica	Peptide-PNA conjugate	[25]
	K. pneumoniae	Peptide-PNA conjugate	[25]
	Shigella flexneri	Peptide-PNA conjugate	[25]
	Listeria monocytogenes	Peptide-PNA conjugate	[26]
ftsZ (cell division)	*P.aeruginosa*	Peptide-PMO conjugate	[15]
	S. aureus	Peptide-LNA conjugate	[27]
		Peptide-PNA conjugate	[28]
rpoA (RNA polymerase sigma factor; transcription)	*L.monocytogenes*	Peptide-PNA conjugate	[26]
argA (accessory gene regulator; virulence regulator; transcription factor)	*S.aureus*	Peptide-LNA conjugate	[29]
carA (amino acid biosynthesis; pyrimidine metabolism)	*A. baumannii*	Peptide-PNA conjugate	[30]

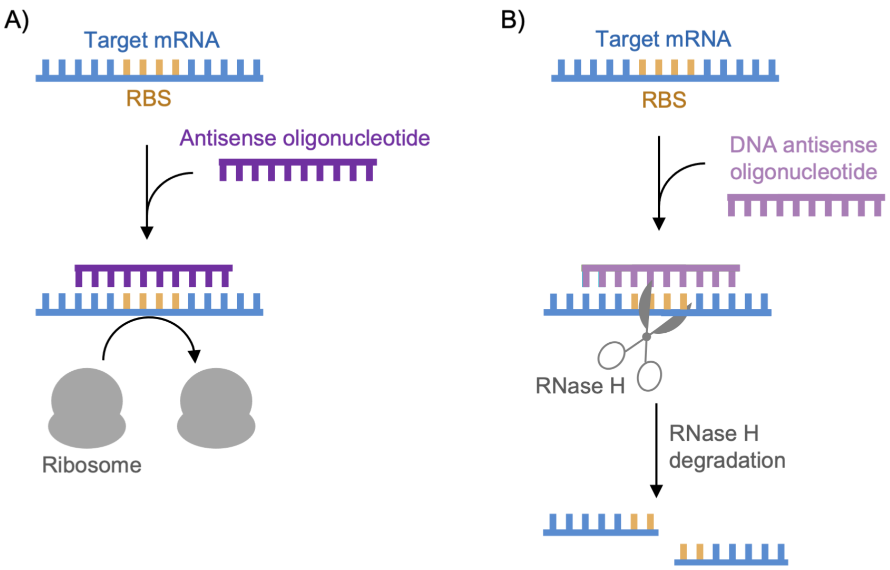

Supplementary Figure S1. Mode-of-action of antisense oligonucleotides. A) Steric blocking of ribosome binding. An antisense oligonucleotide (purple) binds to the translation initiation region (including the ribosome binding site (RBS), gold) of a target mRNA (blue). This prevents the ribosome (grey) from binding and sterically blocks translation. All chemical modifications that are capable of complementary base-pairing with RNA are compatible with this mode-of-action. B) RNase H recruitment and RNase H-mediated mRNA cleavage. A DNA antisense oligonucleotide (mauve) binds to the translation initiation region (including the ribosome binding site (RBS), gold) of a target mRNA (blue). This forms a DNA-RNA hybrid that is a substrate for RNase H (grey scissors). RNase H cleavage results in degradation of the target mRNA which prevents its translation. In order to facilitate RNase H recruitment and mRNA cleavage, the antisense oligonucleotide must contain a region of DNA or modified DNA e.g. phosphorothioate DNA [31].

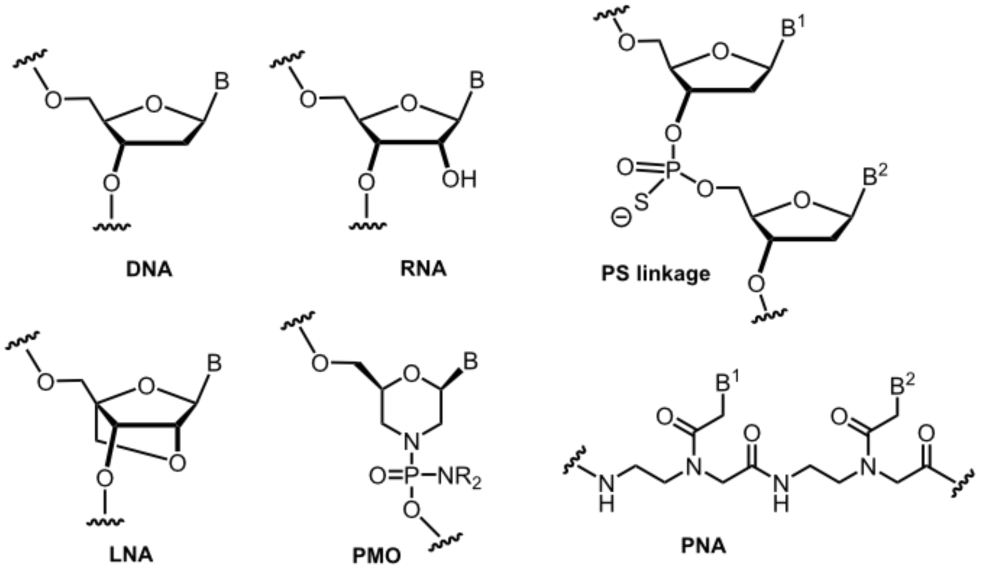

Supplementary Figure S2. Unmodified nucleic acids and examples of chemical analogues used in antisense oligonucleotides. A phosphorothioate (PS) linkage is a backbone modification in which one of the non-bridging oxygens is replaced with sulphur. A locked nucleic acid (LNA) is a sugar modification which reduces the conformational flexibility by covalently linking the 2′-oxygen and the 4′-carbon of the ribose. Both the backbone and sugar have been modified in a phosphorodiamidate morpholino oligomer (PMO). The phosphodiester backbone has been replaced with a non-ionic phosphorodiamidate backbone and the deoxyribose sugar has been replaced with a morpholine ring. The backbone and sugar have also been replaced in a peptide nucleic acid (PNA). In PNA, the phosphodiester backbone has been replaced with an aminoethylglycine backbone and the sugar is replaced with an acetyl linker. $B/B^1/B^2$ are used to indicate the positions of the base(s).

TTGCTATAACAAGGCTTGCAGTGGAATAATGAGGCCGTTTCCGTGTCCATCCTTGTTAAAACAAGAAA
TTTTACGGAATAACCCATTTTGCCCGACCGATCATCCACGCAGCAATGGCGTAAGACGTATTGATCTT
TCAGGCAGTTAGCGGGCTGCGGGTTGCAGTCCTTACCGGTAGATGGAAATATTTCTGGAGAGTAATAC
CCAGTCTGTTTCTTTGATAATTGCGCTGTTTTTCCGCATGAAAAACGGGCAACCGACACTCTGCGCCT
CTTTGAGCTGACGATAACCGTGAGGTTGGCGACGCGACTAGACACGAGGCCATCGGTTCACACCCGGA
AAGGCGTTACTTTGCCCGCAGCTTAGTCGTCAATGTAAGAA**TAATGAGT**AAGTTACG**ATG**AAACGCAT
GCTGATTAATGCAACCCAGGAAGACGCCAAAAACATAAAGAAAGGCCCGGCGCCATTCTATCCTCTAG
AGGATGGAACCGCTGGAGAGCAACTGCATAAGGCTATGAAGAGATACGCCCTGGTTCCTGGAACAATT
GCTTTTACAGATGCACATATCGAGGTGAACATCACGTACGCGGAATACTTCGAAATGTCCGTTCGGTT
GGCAGAAGCTATGAAACGATATGGGCTGAATACAAATCACAGAATCGTCGTGTGCAGTGAAAACTCTC
TTCAATTCTTTATGCCGGTGTTGGGCGCGTTATTTATCGGAGTTGCAGTTGCGCCCGCGAACGACATT
TATAATGAACGTGAATTGCTCAACAGTATGAACATTTCGCAGCCTACCGTAGTGTTTGTTTCCAAAAA
GGGGTTGCAAAAAATTTTGAACGTGCAAAAAAAATTACCAATAATCCAGAAAATTATTATCATGGATT
CTAAAACGGATTACCAGGGATTTCAGTCGATGTACACGTTCGTCACATCTCATCTACCTCCCGGTTTT
AATGAATACGATTTTGTACCAGAGTCCTTTGATCGTGACAAAACAATTGCACTGATAATGAATTCCTC
TGGATCTACTGGGTCACCTAAGGGTGTGGCCCTTCCGCATAGAACTGCCTGCGTCAGATTCTCGCATG
CCAGAGATCCTATTTTTGGCAATCAAATCATTCCGGATACTGCGATTTTAAGTGTTGTTCCATTCCAT
CACGGTTTTGGAATGTTTACTACACTCGGATATTTGATATGTGGATTTCGAGTCGTCTTAATGTATAG
ATTTGAAGAAGAGCTGTTTTTACGATCCCTTCAGGATTACAAAATTCAAAGTGCGTTGCTAGTACCAA
CCCTATTTTCATTCTTCGCCAAAAGCACTCTGATTGACAAATACGATTTATCTAATTTACACGAAATT
GCTTCTGGGGGCGCACCTCTTTCGAAAGAAGTCGGGGAAGCGGTTGCAAAACGCTTCCATCTTCCAGG
GATACGACAAGGATATGGGCTCACTGAGACTACATCAGCTATTCTGATTACACCCGAGGGGGATGATA
AACCGGGCGCGGTCGGTAAAGTTGTTCCATTTTTTGAAGCGAAGGTTGTGGATCTGGATACCGGGAAA
ACGCTGGGCGTTAATCAGAGAGGCGAATTATGTGTCAGAGGACCTATGATTATGTCCGGTTATGTAAA
CGATCCGGAAGCGACCAACGCCTTGATTGACAAGGATGGATGGCTACATTCTGGAGACATAGCTTACT
GGGACGAAGACGAACACTTCTTCATAGTTGACCGCTTGAAGTCTTTAATTAAATACAAAGGATGTCAG
GTGGCCCCCGCTGAATTGGAATCGATATTGTTACAACACCCCAACATCTTCGACGCGGGCGTGGCAGG
TCTTCCCGGCGATGACGCCGGTGAACTTCCCGCCGCCGTTGTTGTTTTGGAGCACGGAAAGACGATGA
CGGAAAAAGAGATCGTGGATTACGTCGCCAGTCAAGTAACAACCGCGAAAAAGTTGCGCGGAGGAGTT
GTGTTTGTGGACGAAGTACCGAAAGGTCTTACCGGAAAACTCGACGCAAGAAAAATCAGAGAGATCCT
CATAAAGGCCAAGAAGGGCGGAAAGTCCAAATTGTAA

Supplementary Figure S3. The *E. coli rne*-firefly *luc* reporter. The DNA sequence for -397 to +30 *E. coli rne* (blue; RBS, gold; start codon, brown) fused to the coding region for the firefly luciferase gene (orange; stop codon, grey).
